# A systematic review of financial incentives given in the healthcare setting; do they effectively improve physical activity levels?

**DOI:** 10.1186/s13102-016-0041-1

**Published:** 2016-06-04

**Authors:** Claudia C. M. Molema, G. C. Wanda Wendel-Vos, Lisanne Puijk, Jørgen Dejgaard Jensen, A. Jantine Schuit, G. Ardine de Wit

**Affiliations:** Department of Tranzo, Scientific Center for Care and Welfare, Tilburg University, PO Box 90153, 5000LE Tilburg, The Netherlands; National Institute for Public Health and the Environment, Centre for Nutrition and Health Services, Bilthoven, The Netherlands; Institute of Resource Economics and Food Policy, University of Copenhagen, Copenhagen, Denmark; Department of Health Science, VU University, Amsterdam, The Netherlands; Julius Center for Health Sciences and Primary Care, University Medical Center Utrecht, Utrecht, The Netherlands

**Keywords:** Financial incentive, Physical activity, Healthcare setting, Systematic review

## Abstract

**Background:**

According to current physical activity guidelines, a substantial percentage of the population in high-income countries is inactive, and inactivity is an important risk factor for chronic conditions and mortality. Financial incentives may encourage people to become more active. The objective of this review was to provide insight in the effectiveness of financial incentives used for promoting physical activity in the healthcare setting.

**Methods:**

A systematic literature search was performed in three databases: Medline, EMBASE and SciSearch. In total, 1395 papers published up until April 2015 were identified. Eleven of them were screened on in- and exclusion criteria based on the full-text publication.

**Results:**

Three studies were included in the review. Two studies combined a financial incentive with nutrition classes or motivational interviewing. One of these provided a free membership to a sports facility and the other one provided vouchers for one episode of aerobic activities at a local leisure center or swimming pool. The third study provided a schedule for exercise sessions. None of the studies addressed the preferences of their target population with regard to financial incentives. Despite some short-term effects, neither of the studies showed significant long-term effects of the financial incentive.

**Conclusions:**

Based on the limited number of studies and the diversity in findings, no solid conclusion can be drawn regarding the effectiveness of financial incentives on physical activity in the healthcare setting. Therefore, there is a need for more research on the effectiveness of financial incentives in changing physical activity behavior in this setting. There is possibly something to be gained by studying the preferred type and size of the financial incentive.

## Background

In high-income countries, 41 % of men and 48 % of women have an inactive lifestyle, based on the World Health Organisation (WHO) Global physical activity guidelines [1, 2]. According to the WHO, physical inactivity is defined as not adhering to physical activity guidelines, thus spending less than 150 min of moderate-intensity aerobic physical activity throughout the week, or less than 75 min on vigorous-intensity aerobic physical activity throughout the week or less than an equivalent combination of moderate—and vigorous-intensity activity [2]. Physical inactivity has negative consequences for people’s health, as it is the fourth leading risk factor for mortality worldwide and it increases the risk of cardiovascular diseases, obesity and diabetes [1–3]. Physical activity can reduce the risk of several chronic conditions, such as diabetes and cardiovascular diseases. Moreover, it is associated with more favorable outcomes in the course of disease. If people would achieve the recommended level of activity, an all-cause mortality risk reduction of almost 30 % would be possible [4]. Still, a substantial proportion of the high-income population is insufficiently active. It is therefore important to find ways to improve physical activity levels, particularly among those who are the least active. However, behavior such as physical activity is complex and therefore difficult to change, implying a serious challenge concerning program adherence and maintaining results after program completion [5, 6].

One setting from which physical activity programs are initiated is the healthcare setting. Many people with (a high risk of) a chronic disease are already within the healthcare setting for treatment of their condition. For these people being physically active to a sufficient extent may be important to prevent a deterioration of their condition. At the same time, healthcare providers can play an important role in motivating patients to participate in a physical activity program [7]. However, research shows that long-term adherence varies greatly between 10 % and 80 % in therapeutic exercise interventions for diabetes patients [8]. There are many reasons that people find it difficult to adhere to exercise schemes, one of which is motivation One of many ways to address motivation is to include financial incentives in the intervention.

Financial incentives provide economic encouragement for people to show desired behavior, such as increasing their physical activity level [9]. Incentives can be either positive or negative. Positive incentives reward individuals either for participation or for when they fulfill the desired outcome of certain health behavior. Negative incentives or disincentives penalize individuals if they do not participate, or if they do not meet the required outcomes established [10].

Financial incentives have the potential to affect both participation rates and program adherence [11, 12]. An important point to address however when studying and discussing effectiveness of financial incentives on behavioral change, is the general notion that a financial incentive constitutes an external motivation for changing behavior. According to the health promotion literature, people need skills and knowledge (intrinsic motivation) to change their lifestyle behavior and simply giving them a financial incentive is not expected to teach them these skills [10, 13, 14]. Building intrinsic motivation takes time and needs work, but financial incentives may help, for instance to increase program adherence to an intervention that teaches these skills and knowledge. Financial incentives can be provided on many levels in healthcare, for example incentives for insurers to promote the financing of exercise programs, for healthcare providers to incorporate physical activity in treatment and rehabilitation, for employers to establish training facilities at work places, or for patients to participate. The providers of the incentives also vary, depending on the healthcare system in a country. Incentives can be provided by the government, insurers, employers or non-profit organizations. The government may have an interest in this, if the benefits to society and/or the government budget (in terms of potential for saved healthcare spending in the long run) exceed the cost of providing the incentive. Similar rationales may apply for insurer—and employer-financed incentive schemes.

Hypotheses on the effectiveness of direct financial incentives to improve physical activity levels vary. One opinion is that offering rewards may be counterproductive in the sense that this extrinsic motivation may crowd out the intrinsic motivation already present. Therefore any increase in physical activity during the time of the intervention, as well as part of the activity level present before the intervention started, will disappear after the incentives are removed [15–17]. A competing hypothesis states that getting people interested in physical activity by giving financial incentives may very well contribute to habit formation. This theory assumes that if exercising is a form of habitual behavior, giving financial incentives to motivate people to exercise for a certain period, may increase future utility from exercising [15, 18]. Previous studies on the effect of financial incentives to change relatively simple health-related behaviors, such as attending appointments at clinics and take up of child immunization, indicate that financial incentives are effective [10, 15]. Systematic reviews on effectiveness of financial incentives to increase physical activity showed positive results in both community- and school setting, particularly in the short term [11, 12]. No such systematic review has been carried out for the healthcare setting. The objective of this study was to systematically review the literature with respect to the effectiveness of direct financial incentives used to promote physical activity in the healthcare setting.

## Methods

### Data sources

A systematic literature search was conducted, using three literature databases (Medline, EMBASE and SciSearch) to find eligible studies on the effect of financial incentives to promote physical activity within a healthcare setting. A combination of search terms covering the healthcare setting (e.g. primary care, delivery of healthcare), financial incentives (e.g. financial support, access and price) and physical activity (e.g. leisure center, active transport) was used to identify all relevant articles (see Appendix 1 for the full search strategy). The search was restricted to publications in English and Dutch and included publications up until April 2015.

### Inclusion and exclusion criteria

**Fig. 1:**
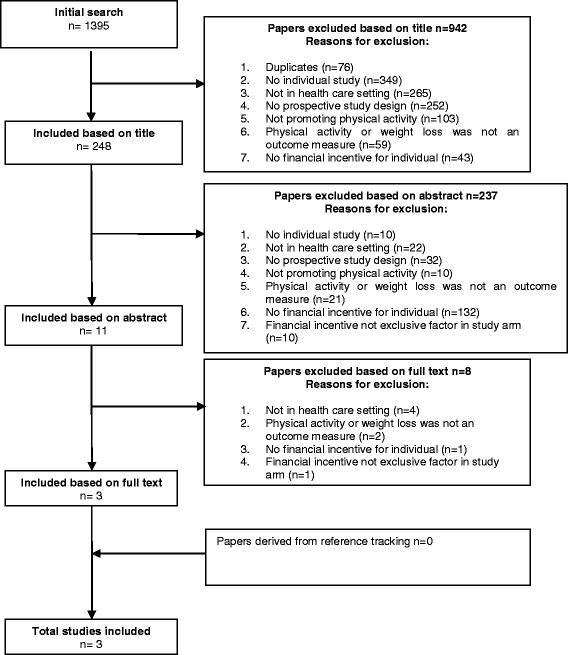
Flow chart describing the systematic search

The primary inclusion criterion was that the paper under consideration had to address physical activity promotion initiated from or within the healthcare setting, including the use of one or more direct financial incentives given to patients. Included studies had to use a prospective design to be able to measure differences over time in individuals and at group level, and provide one or more study arms in which the financial incentive was the exclusive factor, while the goal was to increase people’s physical activity. Effectiveness had to be studied quantitatively in terms of physical activity outcome measures or weight loss. Reviews, editorials and other papers not describing individual studies were excluded. Figure 1 shows the flowchart that contains all exclusion criteria. If one of the criteria was not met, we scored this item a ‘1’. The criteria were scored in a fixed order; if a criterion was scored a ‘1’, assessment of further criteria became redundant.

### Study selection

Publications were selected using a standardized process. Four reviewers (LP, WV, CM and AW) worked in pairs. The first reviewer (LP, CM or WV) selected eligible papers by checking the title against the in- and exclusion criteria and if necessary the process was repeated for the abstract. Another reviewer checked whether the exclusion of the paper by the first reviewer was correct. Any disagreement between reviewers was resolved by consensus. References from the selected full text publications based on their abstract (*n =* 11) were searched for more eligible publications, but did not result in the inclusion of additional publications to be included. Duplicate studies were removed. The process of study selection and reasons for excluding studies are shown in Fig. 1.

### Data extraction

Information was extracted about the first author, year of publication, the setting in which the study was conducted, the study population, description of the intervention and the given incentive, and relevant outcome measures and quantitative results. Table 1 provides a structured overview of the characteristics of the studies included in this review.

**Table 1:** Characteristics and outcomes of the reviewed studies

Author, year	Setting	Study design & study population	Intervention	Outcome measures	Results
Harland et al., 1999 [20]	GP practice in a socio-economically disadvantaged area.	RCT 523 adults aged 40–64 years: C: *n =* 105 I1: *n =* 105 I2: *n =* 106 I3: *n =* 104 I4: *n =* 103	C • Baseline body measurements and information about PA. I1 • Baseline body measurements and information about PA. • Brief motivational interviewing (*n =* 1) during 12 weeks intervention period. I2 • Baseline body measurements and information about PA. • Brief motivational interviewing (n = 1) during 12 weeks intervention period. • 30 vouchers, each for one episode of aerobic activities, at local leisure center or swimming-pool. I3 • Baseline body measurements and information about PA. • Extended motivational interviewing (n = 6) during 12 weeks intervention period. I4 • Baseline body measurements and information about PA. • Extended motivational interviewing (n = 6) during 12 weeks intervention period. • 30 vouchers, each for one episode of aerobic activities, at local leisure center or swimming pool.	• Self-reported physical activity (shortened version of the National Fitness Survey questionnaire).	12 weeks: • No significant effect on PA was found due to the introduction of vouchers or more than one interview. • Significant interaction between providing vouchers and more than one interview: the highest proportion of participants with increased physical activity scores was in the group offered both multiple interviews and vouchers. • Proportion of participants with an improvement on vigorous activity or moderate activity was significantly higher for all intervention groups combined compared to the control group. • No significant effect within the intervention groups due to interviews, vouchers or interactions between them for vigorous or moderate activity. 12 months: • Increases in PA reported at 12 weeks by participants in all intervention groups were not maintained at one year, regardless of the intensity of the intervention.
Duggins et al., 2010 [19]	Family Medicine Clinics and specialized Pediatrics clinics with patients that represented a wide variety of socioeconomic backgrounds.	RCT 83 children aged 5–17 years, with BMI at or above the 85th percentile for age and sex: C: *n =* 39 I: *n =* 44	C • 4 dietician-led nutrition classes (over a 9 months period), discussing diet, nutrition, eating habits and meal planning. In addition, written materials (handbook) were provided. I • 4 dietician-led nutrition classes (over a 9 months period), discussing diet, nutrition, eating habits and meal planning. In addition, written materials (handbook) were provided. • Free 1-year family membership to local YMCA, providing access to all activities, such as swimming, water aerobics, a track for walking or jogging and weights in a variety of sizes. Patients were asked to complete a diary of activities and were reinforced by study staff.	• Year change in BMI-for-age percentile and weight loss	12 months: • No significant differences between groups were found in BMI or change in weight. • The relationship between the number of visits to the YMCA and the loss of either BMI or weight was positive, but very small and not statistically significant.
Islam, 2013 [21]	Rubicon Centre, a facility that provides residential care facility that provides treatment for women with substance abuse disorder	RCT 22 women aged at least 18 years old, who have used cocaine regularly in her lifetime, be approved for 60 days of residential treatment at Rubicon and received medical clearance from the physician to participate: C: *n =* 10 I: *n =* 12	C • Three core exercise sessions scheduled weekly for six weeks, with the opportunity to engage in additional exercise. I • Three core exercise sessions scheduled weekly for six weeks, with the opportunity to engage in additional exercise. • Participants had the opportunity to draw tokens from a prize gym bag if they met the target of 30 min of observed treadmill walking at any intensity. Every time a participant completed the 30 min at a level, she received an escalating number of prize draws. Escalation resumed from baseline (two draws) until the participant completed three consecutive sessions that met the completion of 30 min of exercise criteria. At that time, the number of draws returned to the level achieved prior to reset. Participants received bonus draws if they completed moderate exercise up to 3 times a week.	• Compliance • Anthropometric measurements (BMI and WHR) • Attitudes about exercise (ECS,EBBS and IPAQ-S) • Physical activity levels	6 weeks: • No significant differences were found in minutes spent in exercise sessions, number of completed scheduled 30-min exercise sessions, number of consecutive exercise sessions. • No differences over time were found for both intervention- and control group in BMI and WHR. • No differences over time were found for both intervention- and control group on patients’ attitudes about exercise and in the perception of individuals concerning the benefits of and participating in exercise. • No differences over time were found between intervention- and control group in physical activity levels

Abbreviations used: *BMI* Body Mass Index; *C* control group; *EBBS* Exercise Benefits/Barriers Scale; *ECS* Exercise Confidence Scale; *GP* general practitioner; *I* intervention group; *IPAQ-S* International Physical Activity Questionnaire – Short; *PA* physical activity; *RCT* Randomized Controlled Trial; *YMCA* Young Men’s Christian Association; *WHR* Waist-to-hip ratio

## Results

### Search

In total 1395 papers were found of which 76 papers were duplicates. Based on title and abstract, 1308 publications were excluded. Eleven full-text papers were selected and scored according to the in- and exclusion criteria individually by two reviewers. Finally, three papers, describing randomized controlled trials (RCT) were included (Fig. 1). These studies are summarized in Table 1.

### Study populations, designs and settings

All three included studies describe a RCT. Harland et al. evaluated the effectiveness of several combinations of methods to promote physical activity using brief (one) or extended (six) motivational interviews and a financial incentive for PA promotion (30 vouchers each for one episode of aerobic activities at a local leisure center or swimming pool). This study was performed in the United Kingdom in the primary care setting and involved the local leisure center. In total, 523 adults between 40 and 64 years old were recruited from one urban general practice in a socioeconomically disadvantaged region of Newcastle.

The study of Duggins et al. was designed to address the question, of whether eliminating financial barriers to physically activity leads to weight loss. This study was performed in the USA in the primary care setting in combination with the local Young Men’s Cristian Association (YMCA). In total, 83 children between 5 and 17 years old were recruited in two family medicine clinics and a specialized pediatrics clinic. Patients were eligible if they had a BMI at or above the 85th percentile for age and sex, and the socioeconomic status of the participants varied widely. In the study, participating families were randomized in an intervention group and a control group. Both groups received nutrition advice through four nutrition classes, and to promote physical activity the intervention group received a financial incentive (family membership of the local YMCA). The materials were available in English and Spanish in order to also include Spanish-speaking families.

The study of Islam evaluates a financial incentive in a physical activity program for 22 women of at least 18 years old, who have used cocaine regularly in their lives. The study was performed at Rubcion, a non-profit organization for substance abuse in the USA. Women were eligible if they were approved for 60 days of residential treatment at Rubicon and received medical clearance from the physician to participate. Both groups had an exercise schedule of three weekly sessions for a period of six weeks. In addition, the intervention group had an incentive scheme. If they met their targets in their exercise schedule, participants were allowed to draw tokens from a prize gym bag.

### Financial incentives

All three studies have combined a financial incentive with some other technique, such as motivational interviewing, education or exercise sessions. However, these additional techniques were provided to the individuals in both the intervention group and the control group. As studies were only included in this review when the financial incentive was the only difference between study groups, any effect observed can be assigned to the financial incentive. The incentives in the included studies diverge in their characteristics, such as the value they represent, the requirements to receive the incentive and the moment of handing out the incentive.

Both the studies of Harland et al. and Duggins et al. chose an incentive that is linked to physical activity. The study of Islam chose an incentive in the form of simply a compliment or presents of different values, such as toiletries, jewelry or a digital camera. The higher the value of the incentive, the lower the chance they could grab that prize from the prize gym bag. The study of Islam set requirements in such a way that the participants were only allowed to grab a prize from the prize gym bag if they met their target of 30 min of observed treadmill walking. Some additional prizes could be earned if their adherence to the program was high. In contrast with the study of Islam, the studies of Harland et al. and Duggins et al. did not have requirements that the participants had to meet before they received the incentive.

The studies of Harland et al. and Duggins et al. did not report that the content of the financial incentive was matched with the preferences of the target group. The study of Islam surveyed the participants beforehand and during the intervention to identify which prizes were preferred and whether they were still incentivizing during the intervention. They did not report that they surveyed the preferences for other characteristics, such as the moment of handing out and the requirements for receiving the incentive.

### Study outcomes

Harland et al. evaluated the effectiveness of several combinations of methods to promote physical activity. Data were collected at baseline, at 12 weeks, and after one year. After 12 weeks of intervention, significantly more participants in the intervention group had improved physical activity scores compared to the control group (38 % vs. 16 %, *p =* 0.001). A significant interaction was found between the two intervention conditions (interviews and vouchers) with the greatest effect in the group offered both vouchers and extended interviewing. In general, this pattern was also found when focusing on only vigorous and moderate physical activity. Comparing the matching groups with regard to the number of motivational interviews, no statistically significant effects were found for providing vouchers as a financial incentive as opposed to not providing this incentive. Moreover, effects found at 12 weeks were not maintained one year after the intervention, regardless of the intensity of the intervention. However, the use of vouchers was higher (44 % versus 27 %) among the group that received the intensive intervention (vouchers + six interviews) than in the group that received the brief intervention (vouchers + one interview).

In the study of Duggins no differences in Body Mass Index (BMI) or weight change were seen between the intervention and control group after the one-year intervention period. In the intervention group, the relationship between the number of visits to the YMCA and the loss of either BMI or weight was positive, but very small and not statistically significant.

After the six week intervention period, the study of Islam reported no significant changes over time in both groups for attitude and perception on benefits of participating in exercise, physical activity levels, compliance, BMI, and Waist Hip Ratio (WHR).

## Discussion

The objective of this systematic review was to provide an insight in the effectiveness of financial incentives used for physical activity promotion in the healthcare setting. The search revealed only three eligible studies (two RCTs among adults and one among children) that specifically studied the effect of a financial incentive on improving physical activity measured by physical activity outcomes or weight loss [19–21]. Two of the three studies combined a financial incentive with other methods, such as motivational interviewing or nutrition classes [19, 20]. Despite short-term differences between intervention groups in one study, no differences were found between the control and intervention group over a longer period of time (12 months) in these studies [19, 20]. The study of Islam measured only short term effects and found almost no significant improvements in the intervention group [21]. The included studies do not indicate that financial incentives stimulate physical activity in the healthcare setting.

Two studies included in this review found no long-term effects of the financial incentive. The third study did not measure long-term effects, but did not find important effects in the short term [21]. Harland et al. found some short-term effects. Possibly, the duration and/or intensity of intervention activities in these studies were not enough to alter behavior, since effects regardless of the incentive were small or absent. A well-known physical activity intervention strategy in the healthcare setting is exercise on prescription, which is usually integrated into multidisciplinary combined lifestyle interventions. Such programs tend to include physical activity promotion, improvement of diet, and reduction of psychological barriers using motivational interviewing [22]. Two studies included in this review did not consist of a strong and structured physical activity component, which might have caused participants to focus on other aspects of the intervention than actually becoming physically active [19, 20]. The study of Islam had a structured physical activity component, but the duration was just six weeks [21].

Although the effectiveness of financial incentives on increasing physical activity levels and accomplishing weight loss was generally absent in our review, in other settings, such as the community setting, at least short term effects of financial incentives on physical activity behavior were found [11, 12]. The review of Mantzari et al. has evaluated the effect of financial incentives on health-related behavior, which includes for example healthier eating, physical activity, and smoking cessation. In this review it is also acknowledged that effects are not sustained when the incentive is removed [23].

In all three studies included in our systematic review, a motivation was lacking as to why this particular incentive was chosen for the particular population. It is likely that preferences for a certain type of financial incentive differ between target groups. For example, women may be more risk adverse than men so a financial incentive in the form of a lottery might not be as effective for men as for women [24]. If the specific type of incentive does not fit the preferences of the target population, this may partially explain the lack of its effect on behavior. There is research available that elucidates the importance of some attributes of financial incentives. A broader scoped review on the effectiveness of financial incentives on physical activity showed that for an incentive to be effective it should at least be conditional to the targets set in the intervention [25]. Promberger et al. [26] have performed a discrete choice experiment on the acceptability of financial incentives to change health related behavior. They have found that a preference for the type of incentive for smoking cessation is different than the preferred incentive for weight loss [26]. Moreover, the size of the incentive matters [10] and includes an optimum [27]**.** Therefore, one important recommendation would be to study preferences of the target group to determine a suitable financial incentive before designing and implementing a study.

In a recently published review of reviews the effectiveness of physical activity promotion interventions in the primary care are shown. These interventions seem to have small positive effects [28]. Combining a lifestyle intervention with a financial incentive that is preferred by the target population, might increase the effects on physical activity levels of the individuals. Future research should focus on the most effective combination of the lifestyle intervention and the preferred financial incentive of the target population.

Theoretically, the benefits of the investment in a financial incentive returns to the provider of the incentive, for example in the form of decreased use of healthcare. In national health systems such as in the UK, the provider of the incentive in the healthcare setting is automatically the collector of the benefits. In managed competition systems, insurers might be the provider of incentives with the underlying principle of return on investment, but also gain a competitive advantage in a market with many healthcare insurance providers. It should be acknowledged that financial incentives in the healthcare systems of developing countries might be a bridge too far. The theory of return on investment is a concept that might function as well in healthcare as in the work setting. A review shows that giving incentives in the work setting to employees by providing free wellness programs, and sometimes incentives to increase participation, returns in less healthcare expenditures and less costs for absenteeism [29]. As mentioned before, the present systematic review includes only three studies. We believe however that this is a true reflection of the level of knowledge, despite the fact that the use of financial incentives is fairly common. For example, during many physical activity interventions, participants can freely access sports and/or leisure accommodations or they receive a small reward for participating in the intervention [30, 31]**.** However only a few studies explicitly address the effectiveness of the incentive given in a separate arm of the study, as was one of the inclusion criteria in our study. There were some studies excluded from the review that stated as their aim to evaluate the effect of changing physical activity behavior by giving financial incentives. A closer look at the study methods revealed that this statement could not be justified because of different reasons. These sub-optimal study designs prevented drawing definite conclusions on the effectiveness of financial incentives on physical activity behavior, because for example the effect of the financial incentive could not be distinguished from the other components of the study or the study did not have a control group [24, 30–32].

We decided not to perform a quality check for the included studies. With a yield of only three very diverse interventions addressing the effect of financial incentives on physical activity our review, although systematic in nature, may be characterized as explorative rather than thoroughly addressing the effectiveness of financial incentives in promoting physical activity from the healthcare setting.

One could argue that extending our search with other databases such as EconLit, Psychlit and Sportsdiscus might have increased the yield of the review. However, if we would have missed a key publication, we would have expected it to be found through reference tracking of the studies already included. The limited set of appropriate study designs is confirmed in other systematic reviews. Two other systematic reviews evaluating the effect of financial incentives on physical activity irrespective of the setting included as few as 10 and 11 studies [11, 12]. Moreover, most of the studies included in these reviews defined ‘attendance’ as the incentivized behavior instead of behavioral change. This could also partly explain why few studies are found to be effective in actually changing physical activity behavior. Perhaps incentives may only offer the particular behavior that has been incentivized.

## Conclusion

Few studies have evaluated the effect of a financial incentive on changing physical activity behavior in the healthcare setting. The three studies included in this systematic review did not show effects that could be attributed to the incentive used. However, study designs were not particularly strong and there seems to have been little thought given to whether or not particular incentives suit particular study populations. Nevertheless, based on results in other settings, financial incentives seem promising instruments to increase people’s physical activity.

It is recommended that in future research on the effectiveness of financial incentives on physical activity some basic requirements are met. First, the study protocol should include intervention arms in such a way that effectiveness of incentives can be studied. Second, it is recommended to first study the preferences of the target population with regard to financial incentives to maximize the chance that the incentive will indeed help to increase the intended behavior. Assuming that the control condition will include a program aiming to increase physical activity, it is recommended to consider multidisciplinary combined lifestyle interventions in order to maximize the chance of habit formation and long-term maintenance of behavioral change.

## Abbreviations

BMI, body mass index; C, control group; EBBS, exercise benefits/barriers scale; ECS, exercise confidence scale; GP, general practitioner; I, intervention group; IPAQ-S, international physical activity questionnaire – short; PA, physical activity; RCT, randomized controlled Trial; YMCA, young men’s christian association; WHO, World Health Organisation; WHR, waist-to-hip ratio

## Appendix 1

**Table 2:** Full search strategy

1	(incentive* or reward* or voucher or free access or lottery or lotteries or voucher*1 or prize* or monetary support or financial support or financial assist* or cost sharing or medical fees or subsidy or subsidies or cash payment* or contingent payment* or bonus* or loan* or credit* or member* or financing or disincentive* or penalty or penalties).tw.
2	financial support/or financing, organized/or financing, government/or cost sharing/or fees, medical/or “fees and charges”/or public assistance/
3	(access or participation rate* or “frequency of participation” or sustained participation or increased participation or repeated participation or attendance or (complet* adj3 program) or referral uptake or “used the prescription” or “uptake rate*” or (received adj3 pedometer*) or offered or half price).tw.
4	exercise therapy/ut or “referral and consultation”/ut or counseling/ut or health promotion/ut or health services/ut
5	1 or 2 or 3 or 4
6	(intervention* or program*1 or project*1 or pilot*1 or policy or policies or trial* or increas* or campaign or sustain* or encourag* or motivat* or promot* or improv* or counsel?ing or participation or health facilit*).ti.
7	intervention studies/or health promotion/or health plan implementation/or healthy people programs/or national health programs/or government programs/or program development/or program evaluation/or pilot projects/or exp clincial trials/or counseling/or health facilities/or exercise therapy/or motivation/
8	(excercise referral* or referral program* or exercise program* or excercise promotion or exercise advice*).tw.
9	6 or 7 or 8
10	(physical activit* or exercise or aerobics or aerobic capacit* or aerobic class* or aerobic activ* or physical exert* or moderate activ* or vigorous activ* or sport* or fitness or “keep fit” or gymnas* or gym or walking or walk or running or run or jogging or jog or cycle or cycling or bicycl* or bike*1 or biking or swimming or swim or swims or dancing or gardening or stair*1).ti.
11	(aqua* or yoga* or pilates* or rollerblad* or rollerskat* or skate or skates or skating).ti. or (leisure centre* or leisure center*).tw.
12	(active travel* or active transport* or active commut* or multimodal transportation or alternative transport* or alternative travel* or pedestrianis* or pedestrianiz).ti.
13	motor activity/or exp exercise/or exercise therapy/or exp sports/or fitness centers/or walking/or running/or jogging/or bicycling/or swimming/or dancing/or gardening/or “physical education and training”/or gymnastics/or physical fitness/
14	10 or 11 or 12 or 13
15	9 and 14
16	(health care or health care or primary care or primary health care or preventive care or preventive medicine or health promotion or integrated care or behavi?r therap* or referral scheme* or hospital* or physician* or nurse* or nursing or general practi* or gp or family practi* or doctors or public health).tw.
17	delivery of health care/or delivery of health care, integrated/or primary health care/or preventive medicine/or preventive health services/or primary prevention/or behavior therapy/or hospitals/or physicians/or physicians, family/or physicians, primary care/or family practice/or general practice/or general practitioners/or nursing/or nurses/or “referral and consultation”/or public health/
18	16 or 17
19	5 and 15 and 18
20	(employee* or worker* or work or job or jobs or occupational or school* or pupils or student* or athletes or athletic* or sports medicine or wounds or injuries or injury or incontinence or pregnancy or pregnant or pain or cancer).tw. or injuries.fs.
21	work/or occupational health/or occupational health services/or occupational health physicians/or employee incentive plans/or schools health services/or schools/or students/or student health services/or athletes/or athletic performance/or sports medicine/or exp “wounds and injuries”/or urinary inconticence/or exp pregnancy/or rehabilitation/or exp pain/or pain management/or exp neoplasms/or sports/px
22	19 not (20 or 21)
23	22 and (english or dutch).lg.
24	remove duplicates from 23
